# Electronic Chart Recording for Gamma Knife Stereotactic Radiosurgery

**DOI:** 10.1016/j.adro.2025.101777

**Published:** 2025-05-02

**Authors:** Sven Ferguson, Gregory Kamal, Nels Knutson, Timothy Mitchell, Sreekrishna Murty Goddu, Eric Filiput, Amanda Schoenberger, Joshua L. Dowling, Jiayi Huang, Yi Huang, Taeho Kim

**Affiliations:** a Department of Radiation Oncology, Washington University School of Medicine, St. Louis, Missouri; b Department of Radiation Oncology, Barnes Jewish Hospital, St. Louis, Missouri; c Department of Neurosurgery, Washington University School of Medicine, St. Louis, Missouri

## Abstract

**Purpose:**

Radiation oncology departments are uniquely susceptible to additional challenges when transitioning from paper to electronic chart recording systems. The Gamma Knife stereotactic radiation therapy system has additional complexities stemming from limited network connectivity to other computer systems used within the department. The goal of this project is to transition from paper charting to completely electronic charting.

**Methods:**

To accomplish the digital transformation, our department created a detailed mapping of the paper workflow, converted all documents digitally, implemented a paperless printing solution, and established a digital platform for document processing. Throughout each step, we intended to preserve the integrity of the high-quality treatment workflow in our department by focusing on (1) enhancing the multidisciplinary aspects of the treatment workflow; (2) ensuring protected health information security; and (3) maintaining efficient patient care.

**Results:**

Electronic signature software and a digital printer were installed to overcome technical hurdles. All paper documents, such as the written directive, stereotactic radiosurgery-frame measurements, and physics consult, were converted to electronic documents mainly using spreadsheet applications. Based on clinical implementation and practice, all goals were met which enhanced the treatment workflow by allowing less time spent on documentation and more time with the patients.

**Conclusions:**

With technical planning and coordination from all team members, we demonstrated that the implementation of electronic chart recording systems can be achieved for Gamma Knife radiosurgery treatments that enhance the treatment workflow, provide flexibility for staff, and allow for greater multidisciplinary communication.

## Introduction

Hospital systems in the United States are incentivized to transition from paper charting to electronic charting in part based on the 2009 Health Information Technology for Economic and Clinical Health Act, which provides financial reimbursements for the meaningful use of electronic health records.^1^ This governmental act intended to accelerate the adoption of electronic charting in hospitals given the potential benefits provided by electronic chart recording. Implemented correctly,^2^ hospitals could realize significant cost savings through a reduction in printing costs and decreased employee workloads,^3^ as well as an increase in patient safety, satisfaction, access to information, and quality of care.^4^ Laws and regulations outside the United States may vary, but transitioning to paperless charting should still provide similar benefits. However, given the massive undertaking of the transition some departments, facilities, or even entire hospital systems may be reluctant to make the switch.^5^ Indeed, Middleton et al^6^ found that “balanced against these potential advantages were issues of setup, investment in information technology and staff training and simply the time taken to introduce a truly paperless environment.”^6^ Still, over time, most hospitals systems in the US have gradually switched from paper to electronic record keeping except for specific exceptions that offered their own unique challenges.^7^

### Radiation oncology departments

The paper to electronic charting transition will be different for each department in every institution.^8^ Variability in resources, such as budget and staff, as well as the current extent of digital systems creates a unique environment in which the switch must take place.^9^ Radiation oncology departments in particular exhibit highly intertwined information technology infrastructure, further increasing the complexity. Notable treatment modalities, such as Gamma Knife, present additional and unique complexities. The American Association of Medical Physicists report titled *Electronic Charting of Radiation Therapy Planning and Treatment: Report of Task Group 262* (TG-262) outlines the additional difficulties for nonstandard devices.^10^ The report classifies a standalone as one in which the treatment devices do not connect to any external Radiation Oncology Electronic Medical Records software directly (TG-262 7.1.1 p.e951). One example is the Gamma Knife radiosurgery treatment system. These standalone systems pose a problem when attempting to connect to the established software used in the rest of the department. These systems have limited connectivity because of the lack of bidirectional flow of information, which prevents device control from the Radiation Oncology Electronic Medical Records and direct record keeping. This purposeful disconnect from external electronic devices is partly due to the historical evolution of the systems and partly for network safety. These treatment modalities may have been developed and maintained on more and more independent computer systems, which may have a difficult time connecting to modern reporting systems. For safety reasons, the systems limit interactions with outside networks to ensure the integrity of the safety of the systems.^11^ By restricting the user, the ability to connect to external networks or download files from external devices (eg, USB thumb drive), the risk of malware infection is greatly limited. Because of this lack of connectivity, these treatment modalities commonly use paper charting, even after the remaining of the department may have become completely electronic. The nonintegration of the department’s Radiation Oncology Record and Verify systems led to the manual recording of treatments delivered, lack of control of treatment from the system, and the potential need for additional software solutions between the Radiation Oncology Record and Verify system and treatment systems for robust record keeping, dose composite review, and document completion and signing. These challenges are compounded by the tight regulatory environment in which they operate. Regulated by the Nuclear Regulatory Commission, all processes, procedures, and documentation are subject to high scrutiny through frequent regulatory audits.^12^

### Gamma Knife–specific challenges

Per Nuclear Regulatory Commission regulation 10 code of federal regulations (CFR) 35.40 or agreement states, as well as institutional policy, all plan documents, especially the written directive, must be reviewed, approved, signed, and dated by an authorized medical physicist, an authorized user (radiation oncologist), and neurosurgeon prior to treatment. A paperless process can be implemented to enhance patient care by using a third-party, online, remote digital platform, allowing for expedited documentation processing, and removing potential treatment delays. Under this configuration, although the treatment documentation is being finalized by the physicist, the physicians can focus on other clinical tasks. Other options include using a Radiation Oncology Record and Verify, typically already established within the department, or in-house software created to service the department’s needs.

The Gamma Knife treatment workflow at our facility has a very structured course of care that involves constant communication between multidisciplinary teams. Therefore, throughout the transition, we aimed to maintain the integrity and efficiency of the current treatment process and, if possible, provide additional flexibility to the team. The goal of this manuscript is to document the transition from our institution’s paper workflow into an entirely electronic workflow that may be used as a guide for other institutions looking to do the same. This article is designed to provide insights into the wider process of transitioning from paper to electronic documentation, with a focus on the pros and cons of using a third-party software to establish a connection between standalone systems and established software within the department.

## Methods

To accomplish the desired digital conversion, the following Gamma Knife steps were necessary:

1. Map paper workflow.
2. Convert all documents digitally.
3. Implement paperless printing from the Leksell GammaPlan Treatment Planning System, version 11.3.2, Elekta AB, Stockholm, Sweden.
4. Establish a digital platform for digital document processing.

### Treatment workflow mapping

A successful transition begins by fully mapping out the paper workflow. Workflow mapping begins by defining, at a high level, all major subprocesses that comprise the Gamma Knife treatment workflow. Only minor tasks should be performed outside of these subprocesses. Eventually, it may be found that some tasks do not contribute to the digital workflow and can eventually be omitted for simplicity (eg, transporting the patient from 1 room to another). However, initially, all subprocesses and tasks should be recorded to prevent overlooking important workflow steps.

Once all subprocesses are defined, identify all personnel involved within each subprocess. Next, assign each staff member to a staffing group defined by their clinical role. Finally, document the responsibilities of each role.

The final step in workflow mapping is to identify the documentation used within each subprocess. Understanding each document is fundamental to replicating the data input, signing steps, and handoff of documents in a digital workflow. Detailed information about each document should be recorded, including who initiates the documentation (document origination), all staff interactions with the document, what systems use the data, document handoffs, and who is ultimately responsible for recording the document.

### Paper document digitization

On completion of the treatment workflow map, all documents will have been identified as well as their associated subprocess and users. This information will allow the team to begin the next step: digitizing the paper documents. Each document has varying needs, guiding the choice of digital format. Once this format has been decided for each form, the digital replications can begin. This process includes creating a digital replica nearly exactly as it is in paper form and then modifying the contents as needed. Modifications can range from simple format changes to complete overhauls of the form.

### GammaPlan printing

While investigating the transition plan, a technical challenge presented itself. The Leksell GammaPlan treatment planning system has a unique aspect that requires the treatment plan report to be printed prior to being able to treat the patient. Traditionally, this was not a problem and was solved by simply printing the treatment plan to a printer. However, to make a successful transition to a fully paperless workflow, printing was no longer an option. Attempting a simple “print to PDF” function to bypass this step was unsuccessful due to system configurations; therefore, a more elegant solution was required.

### Establish a digital platform for digital document processing

Eventually, all documentation will need to be gathered to review, obtain signatures of approval, and stored for long-term record keeping. Established software found within the department, for instance, a Record & Verify (R&V) system, may be used if it fits the department’s needs. Optionally, other sources of documentation dissemination and signature acquisition included third-party software options or in-house solutions. Once a digital platform is chosen, the workflow must be replicated within the platform. The depth of this replication will depend on the functionality of the platform itself. Platform capabilities can range from only simple document interactions to automatic routing of documents for review, electronic signatures, enhanced document interactions, and automated documentation markups in the form of templates.

### Time comparison of paper and electronic workflows

To determine whether treatment efficiency is retained within the digital workflow, treatment milestones need to be compared between the paper and electronic workflow. Meaningful data points common across both workflows need to be identified. Timestamps from the treatment planning system, handwritten signatures with date and time, and treatment console provided the most accurate and consistent markers across both workflows.

### Continuation of the integrity of the paper workflow

Throughout each of these steps, the goal was to maintain the integrity of the high-quality paper workflow by focusing on the following aspects:

1. Enhance the multidisciplinary aspects of the treatment workflow.
2. Ensure protected health information (PHI) integrity and security.
3. Maintain efficient patient care.

## Results

### Treatment workflow mapping

**Figure 1:**
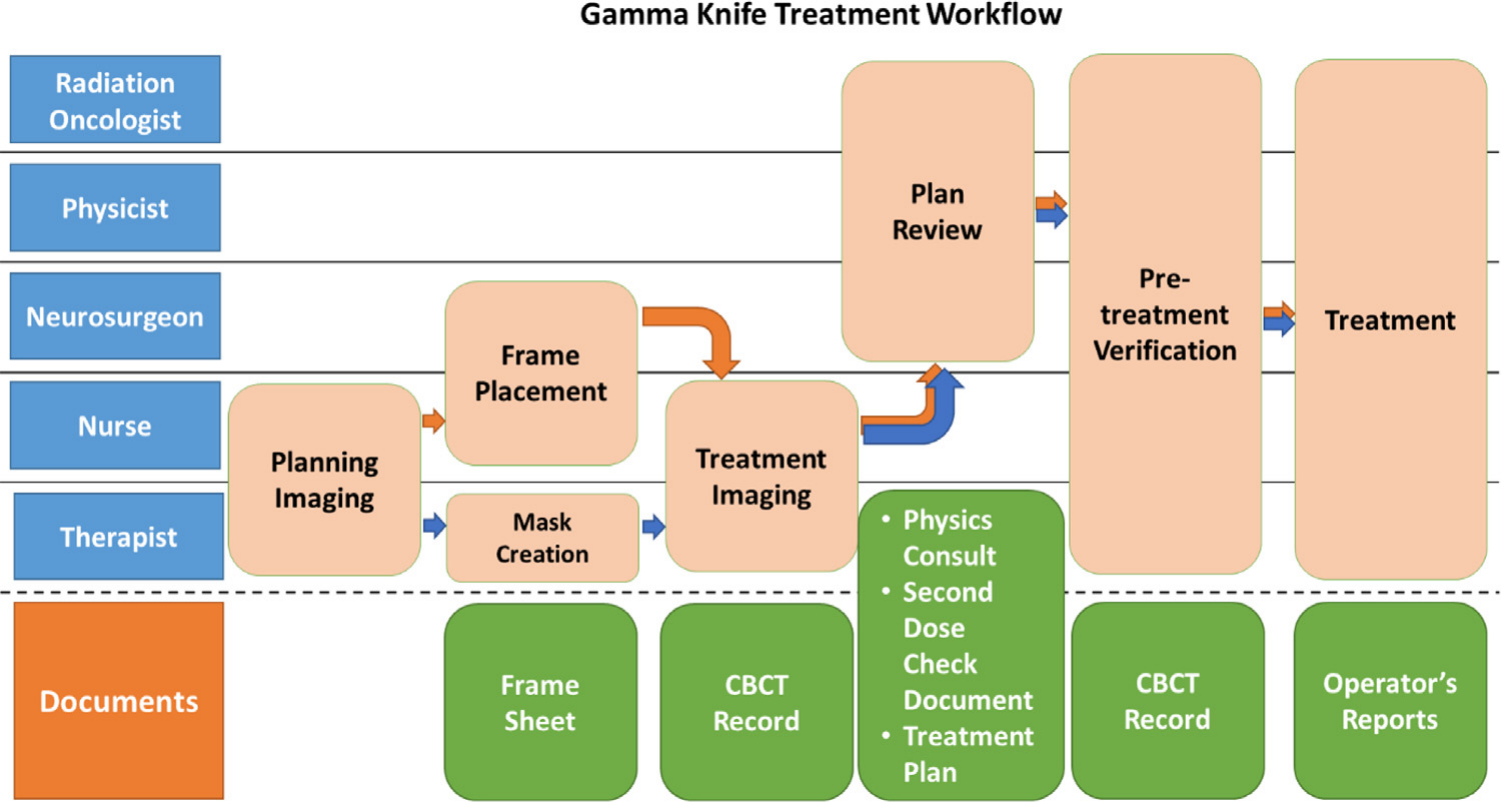
High-level treatment workflow map for framed (orange arrow path) and frameless (blue arrow path) Gamma Knife procedures at our facility. The staff groups involved in each step in the treatment process are listed in the first column, and the documents created within each step are listed in the bottom row. *Abbreviation:* CBCT = cone beam computed tomography.

Our facility has the ability to treat Gamma Knife patients framed or frameless. Figure 1 displays the high-level treatment workflow map for framed (orange arrow path) and frameless (blue arrow path) Gamma Knife procedures at our facility. The 2 workflows follow mostly the same overall paths with slight deviations accounting for either the frame placement or mask creation. The staffing teams who perform the steps, defined in Table 1,^13^ are listed along with the order in which each step will occur. We next identified all documents used within each subprocess, which are listed on the final row of Fig. 1. Table 2 lists all documents generated in the Gamma Knife treatment workflow at our facility along with the users of each document.

**Table 1:** Gamma Knife staffing teams’ roles and responsibilities at our facility

Role	Responsibilities
Medical physicists	Create treatment plans, verify radiation dose accuracy, ensure patient safety
Neurosurgeons	Identify treating target, approve treatment plans
Radiation oncologists	Identify treating target, create and approve treatment plans
Information technology	Provides technical assistance
Nurses^13^	Ensure patient safety and comfort, assist in treatment setup and delivery
Radiation therapists	Ensure patient safety and comfort, create treatment masks, perform treatment setup and delivery

**Table 2:** Documents used in the Gamma Knife treatment workflow at our facility. Users of each document and the electronic document type are also listed

Document	Document type	User
Frame sheet	Spreadsheet	Nurse Physicist
Physics consult form	Spreadsheet	Physicist
CBCT record	Direct to PDF	Therapist
Second dose check documentation	Direct to PDF	Physicist
Treatment plan documentation	Direct to PDF	Nurse Therapist Physicist Radiation oncologist Neurosurgeon
Operator’s report	Direct to PDF	Therapist
Patient treatment note	Word processor	Therapist Radiation oncologist
Frameless fraction form	Word processor	Therapist Physicist Radiation oncologist

### Paper document digitization

The steps involved to converting paper charts to electronic replicas are as follows:

I. Determine each document’s electronic type.
II. Recreate documents digitally.
III. Update and add functionality.

**Figure 2:**
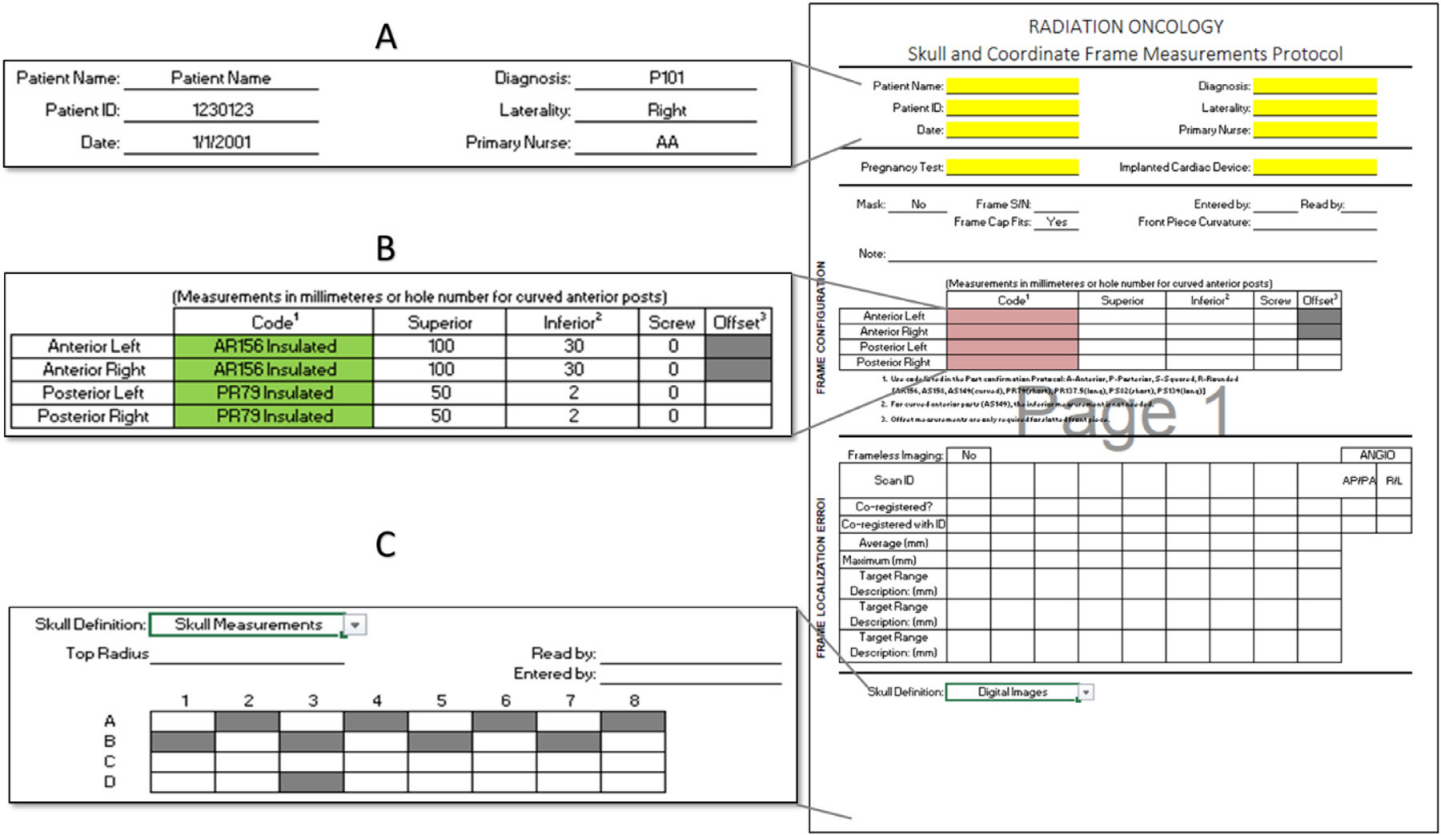
Frame Measurement Sheet for Gamma Knife treatments at our facility. (A) Fields display highlighted yellow until a value is entered. (B) Data validation fields are highlighted red when the entered value is unacceptable and green when acceptable. (C) When Digital Images is selected the entire table is hidden. Selecting Skull Measurements displays the additional fields needed for completion.

**Figure 3:**
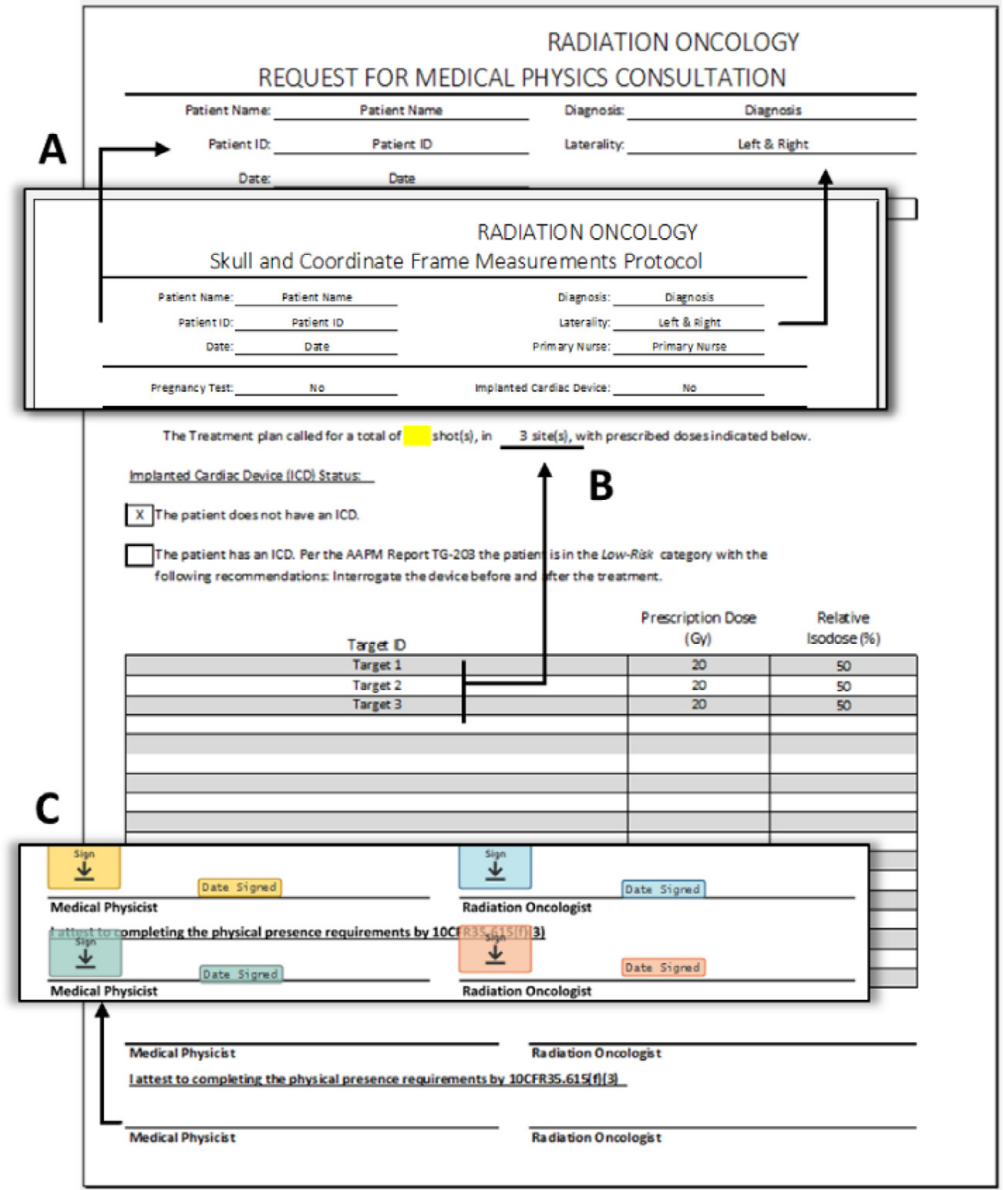
Physics consult document for Gamma Knife treatments at our facility. (A) Data are automatically populated into fields from the Frame Measurement Sheet (Fig. 2). On this form, these fields are locked to retain consistent information across forms. (B) The number of treatment sites updates depends on the number of rows entered into the Target identification (ID) column. Each treatment site has a unique target ID, ensuring accurate treatment site counts using this auto-completion method. (C) The eSignature template application displaying different staff roles across signing steps. Signing is performed before and after treatment by the medical physicist and radiation oncologist.

Our facility found using Microsoft Excel (Microsoft Corporation) provided the best tools for directly interacting with digital documents outside of the digital platform. The 2 documents that were recreated using this spreadsheet software are the frame sheet (Fig. 2) and the physics consult form (Fig. 3). In the clinic, patient data are entered directly into these spreadsheets. Later in the workflow, the spreadsheet is directly added to the digital platform, which converts all the documents to PDF format prior to processing.

Documents that were not generated by another software directly as a PDF (patient treatment note and frameless fraction form) were recreated using Microsoft Word (Microsoft Corporation) and then saved into PDF format. PDFs can have templates applied to them through the digital platform for any further information recording or signatures.

### GammaPlan printing

Through collaboration with our printing vendor (Ricoh Company, Ltd), information technology departments, and Leksell engineers, a solution was created within the Leksell GammaPlan system that simulated a “virtual printer.” By selecting a newly added printer option on the plan report print screen, the system would now intercept the PDF file from the Gamma Knife tool (within the Linux operating system), rename or create a logical file name (via a created script), and then delivered the file to a file or network share drive. This process allowed the plan document to be sent directly to a PDF while simultaneously clearing the system interlock preventing treatment all in 1 step.

### Establish a digital platform for document processing

The commercial software eSignature (DocuSign, Inc) provided the tools needed to process our documents. This online software platform interacts with many different document formats, allowing for the processing of all document types used at our institution. Predefined templates can be applied to the documents for form interaction. All documents can be automatically routed, as a group defined as an “envelope,” providing access to the envelope in a predefined order. Furthermore, this easy-to-use software is accessible without local installation on any web browser or smartphone, enhancing communications with the staff found in Table 1.

The eSignature also provides the required high levels of PHI protection. Documents are first encrypted and then routed through the software to only the intended recipients. Once all documents are completed, they are saved as an unchangeable PDF file protected with a digital certificate that holds records of all transactions during processing. These records include the type of action, timestamps, and location (via internet protocol (IP) address) of the action. These digital certificates are then uploaded directly into our secure R&V software for easily retrievable and detailed record keeping. Additionally, for staff employed by different institutions, who will also have different employee credentials, eSignature’s authorized email approach was of great help by seamlessly incorporating them into the new workflows without the need to create user profiles.

**Figure 4:**
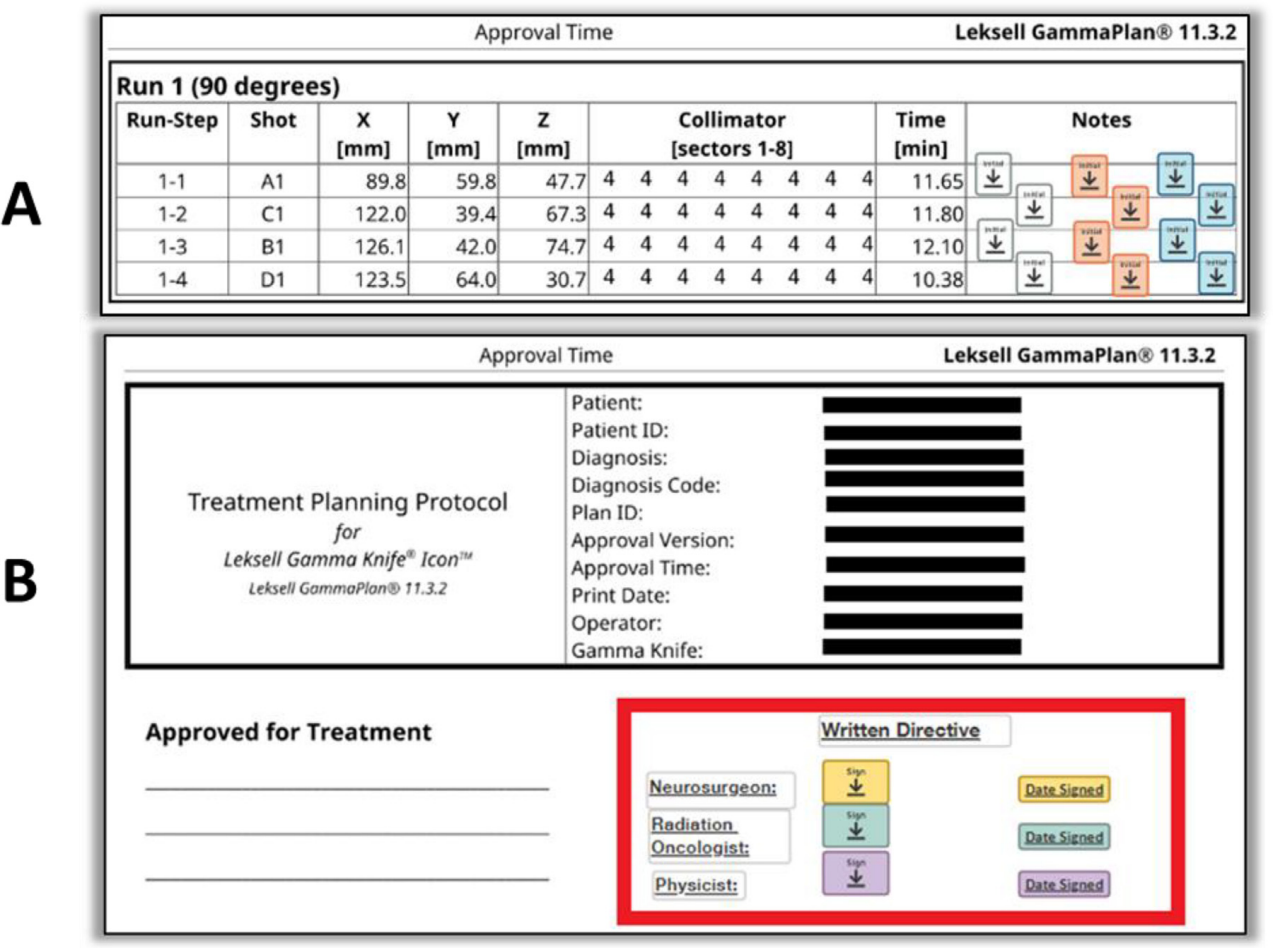
GammaPlan treatment planning documents displaying fields and other markups applied by eSignature’s automatic template recognition. (A) List of shots per target with initial fields for 3 users for each shot. (B) The red box outlines signature and date fields and other markups on the cover page.

We leveraged eSignature’s robust document recognition, template creation, and automatic routing, which provided the time savings and consistency that was key to making the transition work. Using the software tools, digital templates were developed to map to our previous paper-based workflows. The document recognition features allowed auto-population of form fields onto uploaded documents via templates, with automated, predetermined routing at various phases of the treatment process. Figure 3C displays an example of the document recognition and template application. The physics consult form has signature and date and time fields applied in the correct spot automatically. Similarly, Fig. 4 displays the treatment planning document with automatically placed, previously defined markups and signature fields.

**Figure 5:**
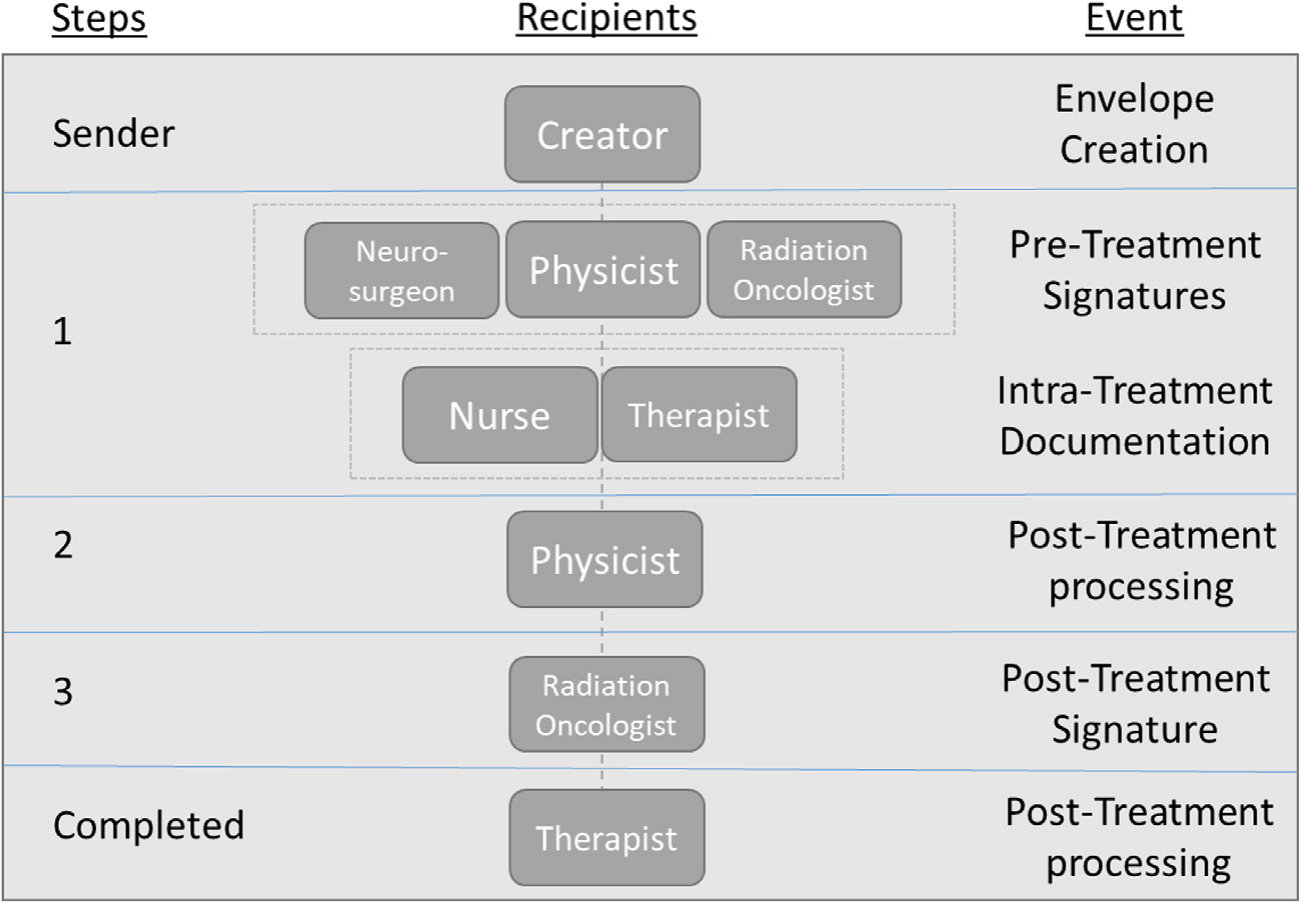
Example of an eSignature envelope, containing treatment documents, which are automatically routed through predetermined treatment steps to predefined recipients. Each step in the signing order coincides with different parts of the treatment process, described by the event column.

Combining steps to single recipients and bridging signing steps across subprocesses improves process efficiency. An example is seen in step 1 of Fig. 5, where our institution combined pretreatment signatures and intratreatment time-outs. Creating the steps in this manner avoids reopening the envelope multiple times, which may cause delays or errors such as 1 user overwriting another.

**Figure 6:**
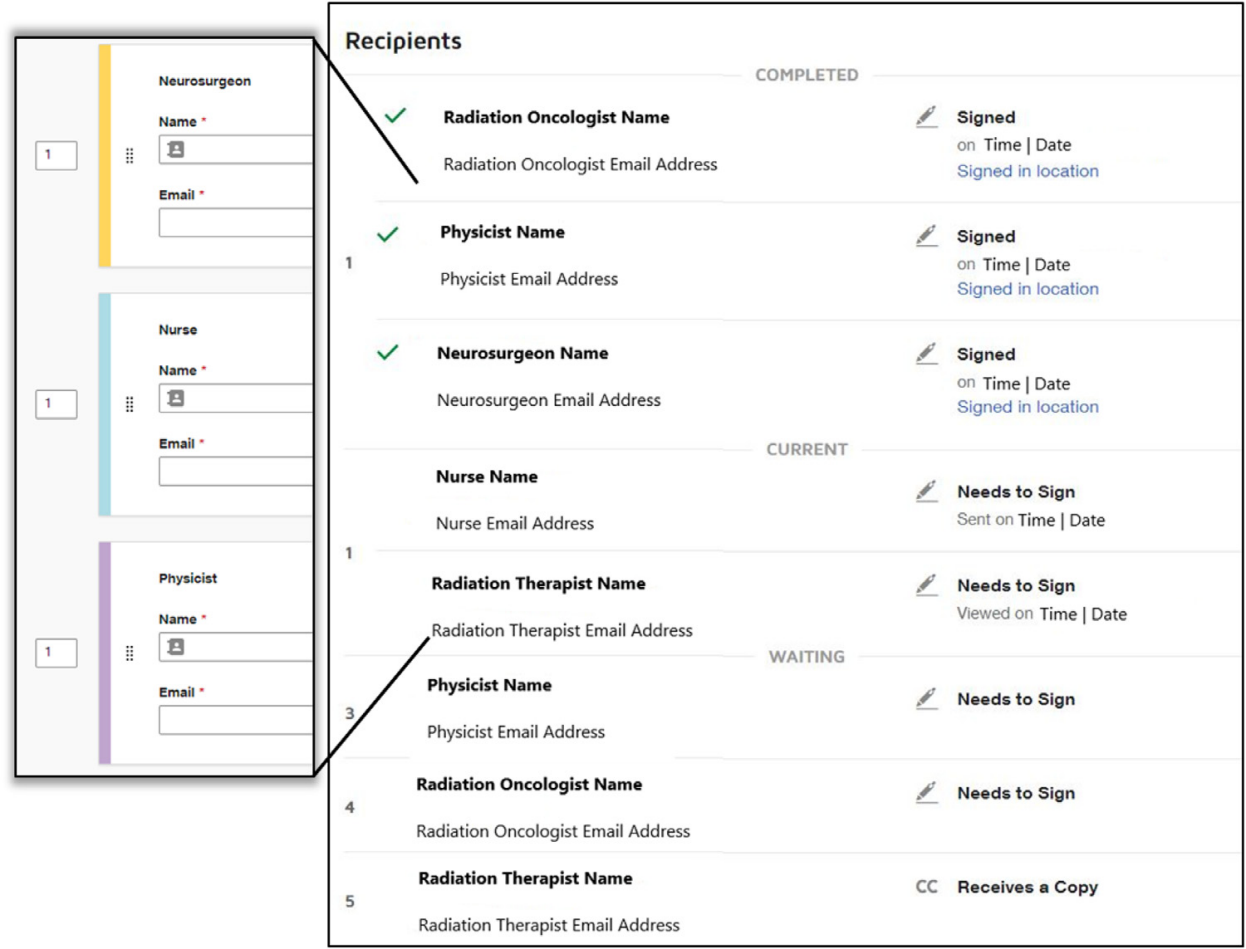
(Left) Example of signing group created for our facility. Each staffing role (Table 1) has a generic signing group. The specific staff member for each role can be assigned just prior to sending the eSignature envelope. (Right) The intended recipients list including the recipient’s name, email address, and status with date and time.

Involving the correct staff at the relevant step can be vastly simplified through the use of templates by having defined signer roles instead of specific staff members. These signer roles, defined in Table 1, were previously identified during the treatment workflow mapping. The specific team member for each role is populated during the document preparation step, which will then be routed to the desired staff at the time of treatment (Fig. 6). Duplicate signing steps for the same role are automatically populated by eSignature once 1 staff member for the role is entered. This process can be simplified further with integrated and bulk-imported email address books. Once a name and email have been entered, they are seamlessly stored in the system for future use.

### Time comparison of paper and electronic workflows

**Figure 7:**
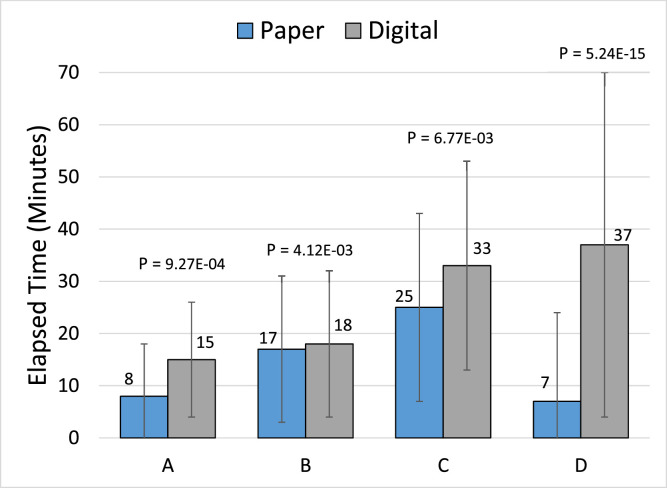
The average elapsed times between various milestones comparing the paper and digital workflows for Gamma Knife treatments at our facility. (A) Plan approval to pretreatment signatures. (B) Pretreatment signatures to treatment start. (C) Plan approval to treatment start, which covers the timeframe spanning both (A + B). (D) Plan completion to posttreatment signatures.

Meaningful data points common across both workflows are pretreatment signatures with timestamps, treatment approval time from the treatment planning system, treatment start time from the treatment console, and posttreatment signatures with timestamps. For paper documents, the written timestamps for pretreatment and posttreatment signatures were tabulated. For electronic documents, the eSignature timestamps for these signatures were recorded. The approval timestamp from the treatment plan documents and the treatment completion documents were used for both the paper and electronic workflows. These data were collected and analyzed from 114 Gamma Knife patients treated with paper charting and 108 Gamma Knife patients treated after the digital conversion. Figure 7 displays these data as the average elapsed times between various milestones in the treatment process. It is shown using the unpaired *t* test that there is a statistically different (*P* < .05) timeframe from plan approval to the start of treatment (Fig. 7C). Additionally, the average time from treatment finish to the final signatures (Fig. 7D) increased with the new workflow. This was expected and did not affect the Gamma Knife patient’s course of care.

### Continuation of the integrity of the paper workflow

The third-party software allowed our center to enhance the multidisciplinary aspects of the treatment workflow by retaining the overall structure of the paper workflow. Electronic documents were substituted for paper documents, with easy access to these documents via computer consoles where needed such as preparatory rooms, treatment planning area, and the treatment room. The PHI integrity and security of this software are in line with the regulatory and departmental requirements. Section “Time comparison of paper and electronic workflows” outlines the expected efficient patient care continued from the paper workflow.

## Discussion

To complete the transition away from paper charting our clinic, we first mapped the paper workflow. During this step, we identified all major subprocesses of the Gamma Knife workflows, all involved staff, and every document used. We then proceeded to recreate these documents digitally, updating them as necessary. Finally, we integrated the documents and workflow within the eSignature digital platform. Once the treatment is complete and all documents are signed, we upload the treatment plan and documents to our department’s R&V for future reference.

To bypass the need to physically print the plan report from the Leksell GammaPlan system, a technical solution was implemented that instead allowed a PDF to be created directly to a location on a network drive. One limitation of this implemented configuration is the location of the saved PDF, which is hardcoded and cannot be changed directly within the GammaPlan printing prompt easily. The initial attempt at PDF printing involved printing flat PDFs, which combine all the document’s layers into 1 single layer. This is the preferred method with patient PHI security in mind, but in our case later posed a problem with implementing document-specific templates in our digital document platform. Although printing to a flattened PDF is common practice for secure documents, a standard PDF was found to be critical for template recognition and workflow optimization.

Cloning paper documents into electronic documents allows for continuity across the transition because the documents will be familiar. Modifications to these documents should be discussed with all users to verify that the changes are prudent. Additionally, some hospital systems have policies that may require document approval from the department for any changes to documents. In these cases, the documents may have to be approved prior to use, which may add more time to the overall transition.

For document handling, currently used software, such as the department’s R&V, is a viable option; however, these systems contained limitations that did not align with our multidisciplinary goals. Physicians and other medical professionals involved in Gamma Knife treatments are already inundated with many hospital computer systems. Requesting these team members, who do not already have access to and are unfamiliar with the R&V, to learn new systems generates frustration leading to transitional resistance. Instead, using the robust, simple, and online eSignature software allows for a seamless transition where team members do not have to install or learn new software. Furthermore, this software is conveniently accessed through several secure methods not provided by our center’s R&V (ie, mobile phones, tablets, or desktop computers), providing great flexibility to team members across departments. Finally, another added benefit of using the eSignature platform is more accurate record keeping. Previously, with paper charting, timestamps would be variable depending on the calibration of the user’s clock. Because all users use the eSignature clock for timestamps, timekeeping is synchronized across users.

In eSignature, we created a single template for each document that is designed to work in tandem with all other templates in complex workflows. To achieve this, it is crucial that templates for each document type accurately map to a specific subprocess in the treatment workflow, involve all relevant staff at their appropriate step in the subprocess, and impose meaningful requirements and restrictions. Features available to electronic forms should be used when prudent. Limits or requirements can allow for the enforcement of legal requirements, whereas routing logic and text-recognition field generation are valuable time-saving features. A well-designed library of templates will improve form standardization, reduce errors in document completion, and limit the need for specialized training specific to their use. In all cases, however, thorough testing is required to ensure features operate as intended in all potential scenarios.

Hospital and university standards for data retention and document security, health insurance portability and accountability act of 1996 (HIPAA) privacy rules, billing and coding requirements, and Nuclear Regulatory Commission Guidelines are all considerations when making the transition to a paperless workflow. Any software that could record signature approvals needs to be vetted by the administrative legal team. Additionally, any digital document handling would need to be analyzed to verify HIPAA compliance with appropriate document security. Finally, establishing a partnership with a new vendor requires contract review and negotiation on pricing. All these steps may take an extended period of time to complete, potentially delaying the implementation of the digital transition.

Clinical data from other institutions are accepted in either (1) digital dmaging and communications in medicine (DICOM) format or (2) documentation if DICOM information is unavailable. For every Gamma Knife treatment and other external beam treatment modalities, as well as outside records, DICOM data are uploaded to our departmental external treatment planning system (TPS) for record keeping and dose composite information. Generally, outside organ at risk (OAR) and target nomenclature are not reviewed because we tend to focus on regions of high-dose overlap instead.

One limitation of this transitional approach is the added financial and staffing cost associated with the new software. The initial purchase and monthly usage costs will vary per institutional contract but may be a limiting factor for some facilities that may not be able to justify the additional costs. Similarly, the staffing needs may prohibit some centers’ from attempting this approach. General staffing needs for this transition include at least 1 person who is familiar with the process from beginning to end and can be available as needed, which can be time-consuming. Other involved staff will need to be available as needed for review of the implementation plan and requested changes. This new process does add on average extra time from treatment completion to the final signatures (as seen in Fig. 7D) providing the physicist more time to thoroughly review all documents for accuracy prior to the final signatures. Under the prior workflow, the physician was required to remain physically present, while all documents were reviewed and prepared for the final signatures. Given this constraint, these documents were more quickly prepared and presented to the physician for signatures. Under the new process, the physician can provide various other patient care during this preparation time and sign the documents remotely at their convenience using the new digital platform. Because the treatment is already complete, this timeframe does not impact patient treatment and provides for more accurate treatment records. Figure 7 displays a slightly longer average preparatory time between the plan approvals (Fig. 7A) which most likely stems from the extra time needed to gather, process, and send out the documents digitally.

Once fully transitioned, the “go-live” date was chosen to be a day with the minimum caseload of 1 patient. This allowed the team to move carefully through each step of the process without the burden of added pressure to quickly service multiple concurrent patients. Everyone was trained and alerted to the transition plan in advance, so they were prepared to use the new workflow. In the event an unsurmountable issue arises with the new digital process, the backup plan is to revert to paper charting until the issue is resolved. By retaining the structure of the previous workflow, with electronic documentation substituting paper, our center was able to continue the efficiency of the Gamma Knife treatment workflow to which our center has become accustomed. The eSignature provides the added flexibility of remote reviewing, approving, and signing options, thereby enhancing the previous workflow. Additionally, all the security of the new process is in line with regulatory and departmental requirements. All these changes combined provide our center to continue the efficient patient care that was established under the previous workflow.

## Conclusion

Through proper planning and dedication, standalone systems, such as the Gamma Knife, even with their additional challenges, can successfully transition from paper to fully electronic chart recording. Our institution was able to make this transition in the Gamma Knife treatment workflow with little to no disturbance to patient treatment. Successful implementation of electronic chart recording systems can allow for the removal of all paper charting while enhancing the workflow of a standalone treatment facility, such as the Gamma Knife system and other similar systems.

## Disclosures

The authors declare that they have no known competing financial interests or personal relationships that could have appeared to influence the work reported in this paper.
